# The Role of Nurse Managers in Promoting Psychological Safety and Patient Safety Culture in Nursing Teams: A Scoping Review

**DOI:** 10.3390/healthcare14162590

**Published:** 2026-08-18

**Authors:** Cristina Augusto, Soraia Pereira, Daniel Cunha, Inês Rocha, Maria José Lumini, Renata Santos, Patrícia Gonçalves

**Affiliations:** 1 RISE-Health, Nursing School, University of Porto, Rua Dr. António Bernardino de Almeida 830, 4200-072 Porto, Portugal; cristinaaugusto@esep.up.pt (C.A.); danielcunha@esep.up.pt (D.C.); inesrocha@esep.up.pt (I.R.); lumini@esep.up.pt (M.J.L.); patriciagoncalves@esep.up.pt (P.G.); 2 Santo António Local Health Unit, Largo Professor Abel Salazar, 4099-001 Porto, Portugal; rsantos.27.rs@gmail.com; 3 Santa Maria Health School, Tv. de Antero de Quental 173 175, 4049-024 Porto, Portugal

**Keywords:** psychological safety, patient safety culture, nurses, nurse manager

## Abstract

**Background/Objectives:** Psychological safety is increasingly recognized as a key factor in promoting communication, learning, and teamwork in healthcare, with implications for patient safety culture. Although psychological safety and patient safety culture have been reviewed separately, evidence on how nurse managers contribute to both constructs within nursing teams remains fragmented. This scoping review aimed to map the available evidence on the role of nurse managers in promoting psychological safety and patient safety culture in nursing teams, identify leadership behaviors, strategies, and competencies, and highlight facilitating factors and barriers. This review provides an integrated synthesis of evidence on how nurse managers contribute to fostering both psychological safety and patient safety culture within nursing teams, addressing a gap in the existing literature. **Methods**: A scoping review was conducted following established methodological frameworks and prospectively registered in the Open Science Framework. Systematic searches were conducted in MEDLINE (PubMed), CINAHL, Psychology and Behavioral Sciences Collection, Scopus, BVS, and WorldCat for gray literature, with the final search performed on 11 May 2026. A total of 229 records were identified, with 112 duplicates removed. The remaining 117 records were screened, resulting in 25 studies included for analysis. Data were extracted and synthesized using thematic analysis. **Results**: Twenty-five sources were included, comprising predominantly quantitative (mainly cross-sectional) studies, together with qualitative studies, systematic reviews, conceptual papers, expert opinion papers, and gray literature. The findings highlight a close and interdependent relationship between psychological safety and patient safety culture. Nurse managers’ relational leadership behaviors—such as visible presence, active listening, emotional support, and constructive feedback—emerged as key strategies for fostering psychologically safe environments. The reviewed evidence suggests that psychological safety may mediate the relationship between leadership practices and communication, speaking up, incident reporting, and organizational learning. Facilitators include supportive leadership and a just culture, while barriers encompass punitive environments, hierarchical structures, workload pressures, and resource constraints. **Conclusions**: The findings suggest that psychological safety may be an important mechanism through which nurse managers contribute to patient safety culture. However, leadership alone is insufficient, requiring alignment with organizational conditions and system-level support. These findings support strategies to strengthen safety culture and guide leadership development.

## 1. Introduction

Psychological safety has gained increasing attention in healthcare due to its association with team functioning, organizational learning, professional well-being, and patient safety. Edmondson defined psychological safety as “a shared belief held by members of a team that the team is safe for interpersonal risk taking” [1]. Building on this conceptualization, the core attributes of psychological safety have been identified as perceptions regarding the consequences of interpersonal risk-taking, strong interpersonal relationships, the group-level nature of the construct, the existence of a safe environment for interpersonal risk-taking, and a non-punitive culture [2]. In psychologically safe environments, healthcare professionals feel able to ask questions, seek help, admit mistakes, voice concerns, and report errors without fear of embarrassment, rejection, or punishment [1]. Such conditions are particularly important in healthcare settings, where effective care delivery depends on communication, collaboration, and continuous adaptation to complex clinical situations [1,2]. In healthcare, psychological safety is associated with open communication, error reporting, collaborative practice, and learning-oriented behaviors, all of which contribute to improvements in care quality and safety [2,3].

While psychological safety focuses on team members’ perceptions of interpersonal risk-taking, patient safety culture represents the shared values, attitudes, competencies, and behavioral patterns that determine an organization’s commitment to safety and its ability to manage safety effectively [4,5]. It has become a strategic priority for healthcare organizations, given the recognized impact of safety culture on adverse events, organizational learning, and quality of care [2,5]. Key dimensions of a positive patient safety culture include leadership support, communication openness, non-punitive responses to error, teamwork, and organizational learning [4,6].

The relationship between psychological safety and patient safety culture has been increasingly recognized in the literature [3,7]. Psychological safety facilitates behaviors that are essential for safety culture, including speaking up about concerns, reporting incidents, sharing knowledge, and engaging in collective learning [4]. Consequently, it has been described as an important mechanism through which healthcare organizations can strengthen both patient safety outcomes and professional well-being [8].

Although closely related, it is important to distinguish between psychological safety, patient safety culture, and nurse manager leadership, which represent distinct but complementary constructs. As noted above, psychological safety concerns the interpersonal climate within a team, whereas patient safety culture reflects the broader organizational environment in which team-level behaviors are encouraged, reinforced, and sustained [3]. Nurse manager leadership constitutes a key organizational influence that connects these two constructs by fostering supportive relationships, promoting communication, encouraging learning from errors, and modeling behaviors consistent with a just, non-punitive culture [6,9,10]. As formal leaders responsible for coordinating nursing teams, organizing care delivery, and translating organizational priorities into everyday clinical practice, nurse managers are uniquely positioned to strengthen both psychological safety and patient safety culture [6,8]. Through leadership behaviors characterized by visibility, inclusiveness, support, constructive feedback, and responsiveness to staff concerns, nurse managers create conditions that enable healthcare professionals to engage in interpersonal risk-taking while simultaneously contributing to the development of safer organizational cultures [6,9]. Understanding these distinct yet interconnected concepts is therefore essential for explaining how nursing leadership contributes to safer, learning-oriented healthcare environments.

In practice, this leadership role is operationalized through everyday interactions with frontline staff. Nurse managers can promote communication openness, encourage incident reporting and learning, support staff well-being, and foster a just and non-punitive culture, thereby reinforcing both psychological safety and a positive patient safety culture [4,11]. By creating opportunities for open dialog and continuous learning, nurse managers contribute to environments where staff feel empowered to raise concerns, participate in quality improvement initiatives, and engage in collective learning processes that ultimately strengthen patient safety [10,11].

Nevertheless, the development of psychologically safe and safety-oriented work environments remains challenging. Barriers such as punitive responses to incident reporting, staffing shortages, excessive workloads, limited organizational support, and inadequate feedback mechanisms may hinder communication, learning, and safety improvement efforts [7,12]. Understanding how nurse managers address these challenges is therefore essential to strengthening both psychological safety and patient safety culture within nursing teams.

Despite the growing body of research on psychological safety, patient safety culture, and healthcare leadership, evidence regarding the specific contribution of nurse managers remains fragmented [3,8,9,13]. Previous evidence syntheses have examined these topics from different perspectives but have not addressed their integration within nursing leadership. For example, evidence synthesized across healthcare settings has highlighted the relevance of psychological safety to communication, learning, and staff experiences, but without specifically examining the role of nurse managers or its relationship with patient safety culture [12]. Similarly, interventions aimed at improving patient safety culture among healthcare workers have been reviewed, but without focusing on nurse manager leadership or exploring psychological safety as a mechanism through which safety culture may be strengthened [7]. Consequently, evidence regarding how nurse managers simultaneously contribute to fostering psychological safety and patient safety culture within nursing teams remains dispersed across the literature. By integrating psychological safety and patient safety culture through the specific lens of nurse manager leadership, this review addresses an important gap in current knowledge and provides a more comprehensive understanding of how nurse managers contribute to safe and learning-oriented nursing work environments.

Given the exploratory nature of the review question and the conceptual and methodological heterogeneity of the available evidence, a scoping review approach was considered the most appropriate methodological framework. The literature encompasses a range of study designs, leadership frameworks, and understandings of psychological safety and patient safety culture. As such, a scoping review is particularly suited to mapping the breadth of evidence, identifying key concepts, and highlighting knowledge gaps in complex and evolving fields.

Accordingly, this scoping review aims to map the available evidence on the role of nurse managers in promoting psychological safety and patient safety culture in nursing teams. Specifically, it seeks to identify the leadership behaviors, strategies, competencies, facilitating factors, and barriers associated with nurse managers’ contributions to psychologically safe and safety-oriented nursing work environments.

## 2. Materials and Methods

This scoping review was conducted in accordance with the Joanna Briggs Institute (JBI) methodology for scoping reviews [14], and the reporting followed the Preferred Reporting Items for Systematic Reviews and Meta-Analyses extension for Scoping Reviews (PRISMA-ScR) guidelines [15]. The review was previously registered in the Open Science Framework to ensure methodological transparency and the reproducibility of the research process and to reduce the risk of bias [16].

### 2.1. Eligibility Criteria

The Population–Concept–Context (PCC) framework was used to define the eligibility criteria [17]. The population of interest included nurse managers, nurse leaders, and other nursing leadership roles involved in the supervision, coordination, or management of nursing teams. The central concepts were psychological safety and patient safety culture, including related constructs such as open communication, speaking up, error reporting, team learning, and leadership behaviors that foster safe team environments. The context included all healthcare settings where nursing teams operate, such as hospitals, primary care, long-term care, and other clinical or organizational environments. The criteria and operational definitions are summarized in Table 1.

Eligible sources included quantitative studies, qualitative studies, mixed-methods studies, observational designs, interventional studies, and systematic reviews that addressed the role of nurse managers in promoting psychological safety and/or patient safety culture in nursing teams. Published and unpublished studies in any language were considered, with no time restrictions applied. Editorials and conference abstracts were excluded. Conceptual papers, expert opinion papers, and relevant gray literature were eligible when they addressed the review question.

### 2.2. Search Strategy

A three-step search strategy was implemented to identify relevant evidence [14,17]. First, an initial limited search of MEDLINE (PubMed) and CINAHL (EBSCO) was undertaken to identify key terms, subject headings, and index terms related to nurse managers, psychological safety, patient safety culture, and nursing teams. The text words found in titles and abstracts of relevant articles, together with database-specific indexing terms, informed the development of the full search strategy.

Second, the refined search strategy was applied on 11 May 2026, across MEDLINE (PubMed), CINAHL (EBSCO), Psychology and Behavioral Sciences Collection (EBSCO), Scopus, and BVS. WorldCat was searched to identify gray literature, including theses, dissertations, reports, and other relevant non-indexed sources.

Third, the reference lists of all included studies were screened manually to identify additional eligible sources. Searches were not limited by publication date or language. Both published and unpublished evidence were included to maximize sensitivity and ensure a comprehensive mapping of the available literature. The full search strategy was adapted to the indexing rules of each database. Both controlled vocabulary terms (e.g., MeSH terms and equivalent indexing terms, where available) and free-text keywords were combined and tailored to each database to maximize sensitivity and comprehensiveness. The complete search strategies are provided in Supplementary Material Table S1.

### 2.3. Study Selection

All identified records were imported into a reference management system, and duplicates were removed before screening. Title and abstract screening were conducted independently by two reviewers, followed by full-text assessment of potentially relevant studies. A pilot screening of the initial set of titles and abstracts was first performed to ensure consistency in applying the eligibility criteria. Any disagreements between reviewers were resolved through discussion and consensus. Although study selection was conducted independently by two reviewers, inter-reviewer agreement statistics (e.g., Cohen’s kappa) were not calculated. Studies were included if they explicitly addressed nurse managers’ or nursing leaders’ role in promoting psychological safety and patient safety culture. Studies that focused exclusively on general patient safety without reference to leadership, team climate, or psychological safety were excluded. Likewise, studies centered solely on individual clinician behavior without relevance to nurse management were not retained.

### 2.4. Data Extraction and Synthesis

Data extraction was performed independently by two reviewers using a structured chart developed by the authors for this review. The extraction form was designed to capture the following information from each included study: title, author(s) and year of publication, country of origin, context, type of study, and goal(s). In addition, the form included study findings related to the role of nurse managers in promoting psychological safety in nursing teams, the role of nurse managers in promoting patient safety culture in nursing teams, the relationship between psychological safety and patient safety culture, and the facilitating factors and barriers influencing nurse managers’ performance in promoting these outcomes.

An inductive thematic synthesis approach was used to analyze the extracted findings. Codes were generated directly from the data, allowing themes to emerge from the included evidence rather than being predefined. Similar codes were subsequently grouped into broader thematic categories through iterative comparison, discussion, and refinement among the review team until agreement was reached on the final thematic structure.

Systematic reviews were included as sources of evidence and synthesized alongside primary studies. The thematic synthesis focused on concepts, findings, and themes reported across the included evidence rather than on quantitative aggregation of primary studies. Consequently, potential overlap of primary studies across included reviews was not considered to affect the thematic mapping objectives of this scoping review.

This structured approach allowed for consistent mapping of the evidence and supported the subsequent descriptive and thematic synthesis of the findings. It also enabled comparison across studies according to the review question and objectives, while ensuring that both conceptual and contextual aspects of nurse managers’ contribution to psychological safety and patient safety culture were systematically captured.

### 2.5. Critical Appraisal

Consistent with JBI guidance for scoping reviews, no methodological quality assessment of the included evidence was undertaken. The purpose of this review was to map and characterize the available evidence rather than to evaluate the methodological quality or certainty of the findings.

## 3. Results

**Figure 1:**
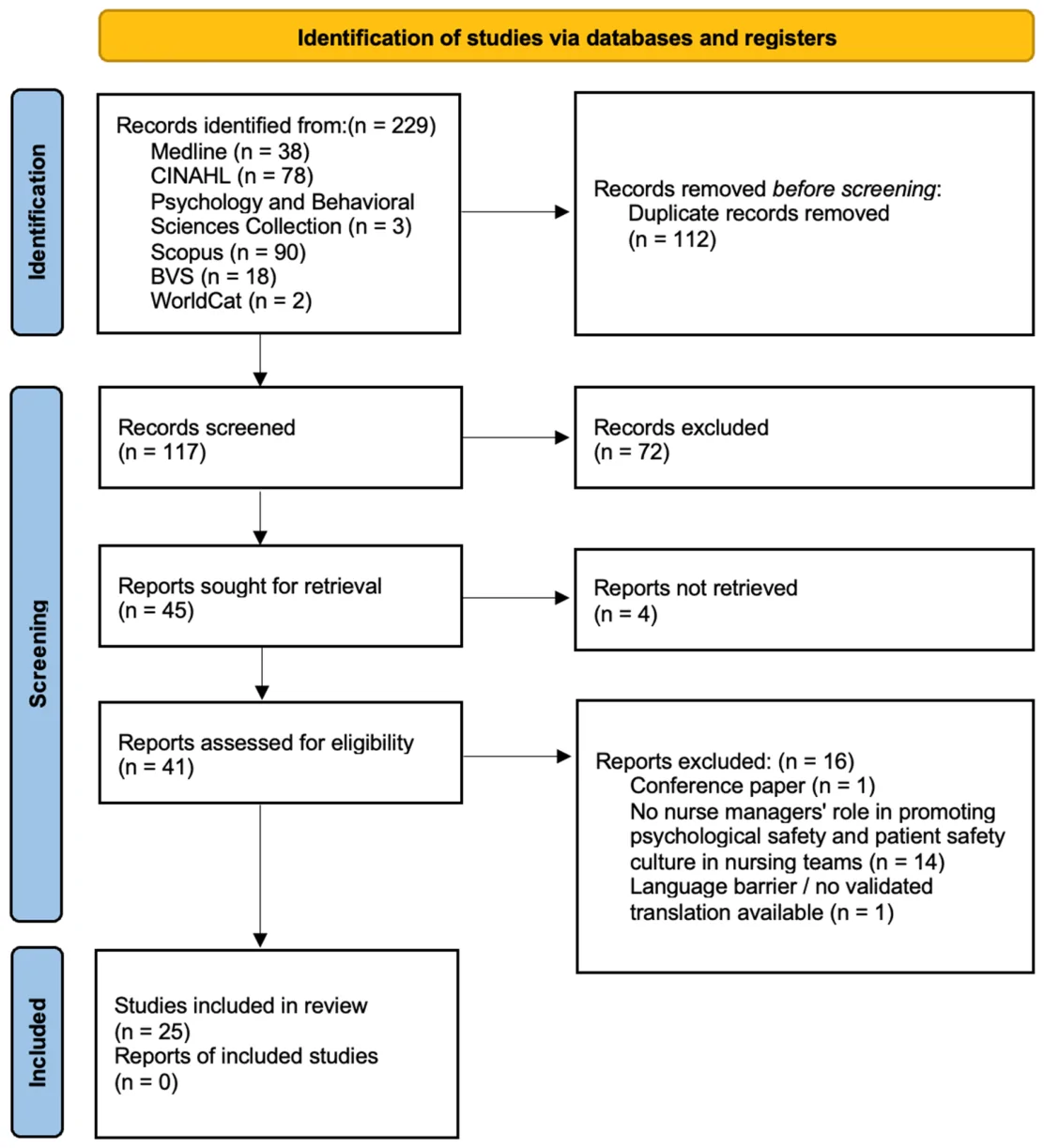
PRISMA flow diagram of study selection process (adapt. from Page et al., 2021 [19]).

The study selection process is summarized in the PRISMA 2020 flow diagram (Figure 1). Database and register searches identified 229 records. After the removal of 112 duplicates, 117 records underwent title and abstract screening, of which 72 were excluded. Forty-five reports were sought for retrieval, four of which could not be retrieved. Consequently, 41 full-text reports were assessed for eligibility. Sixteen reports were excluded following full-text review, including one conference paper, 14 that did not address the role of nurse managers in promoting psychological safety and patient safety culture in nursing teams, and one that could not be assessed because no validated translation was available. Overall, 25 studies met the eligibility criteria and were included in this scoping review. The included publications were published between 2008 and 2026 and reflected methodological diversity. Seventeen studies were published from 2021 onwards, corresponding to 68% of the sample.

The included literature comprised a range of publication types and study designs. Most publications were quantitative studies (*n* = 14), predominantly cross-sectional. The review also included qualitative studies (*n* = 5), reviews (*n* = 3), conceptual or expert opinion papers (*n* = 2), and one doctoral thesis comprising a systematic review, a conceptual paper, and quantitative empirical studies. In terms of country of origin, most publications originated from the United States (*n* = 12), followed by South Korea (*n* = 5) and China (*n* = 2). The remaining publications originated from Israel, Canada, the Netherlands, Ireland, Saudi Arabia, and Belgium (*n* = 1 each). This geographical distribution indicates that the available evidence is concentrated in a limited number of countries, particularly the United States and South Korea. Consequently, differences in healthcare systems, organizational structures, nursing roles, and cultural contexts should be considered when interpreting the findings and assessing their transferability to other healthcare settings.

Most studies were conducted in hospital settings, particularly acute care hospitals, with many focusing on inpatient units, including medical–surgical wards and intensive care units. Other healthcare settings were less frequently represented. Overall, the included studies focused on nursing teams and healthcare environments in which nurse manager leadership, psychological safety, open communication, speaking up, error or adverse event reporting, and patient safety culture were central concerns (see Table S2).

The thematic synthesis of the included evidence identified seven interrelated dimensions concerning the role of nurse managers in promoting psychological safety and patient safety culture in nursing teams. These dimensions were: the role of nurse managers in promoting psychological safety; the role of nurse managers in promoting patient safety culture; the relationship between psychological safety and patient safety culture; speaking up as a behavioral mechanism; facilitators; main barriers; and knowledge gaps and future research needs.

Across the included studies, terminology varied, with some studies referring to nurse managers, nurse leaders, nursing leaders, supervisors, or managers. For consistency, this synthesis uses nurse managers as the primary term, while recognizing that some evidence refers more broadly to nursing leadership roles.

Overall, the findings showed considerable convergence across the included studies. Nurse managers were consistently described as key actors in creating psychologically safe work environments, strengthening open communication, supporting learning from errors, and promoting safety-oriented nursing practice. Table 2 presents the thematic synthesis of the extracted evidence.

### 3.1. Role of Nurse Managers in Promoting Psychological Safety

The first dimension concerned the role of nurse managers in promoting psychological safety. The evidence indicates that nurse managers contribute to psychologically safe environments through visible leadership [28,31,35], active listening [20,33,35,37,39], respectful responses [20,33], emotional support [20,24,25,27], modeling of safe behaviors [24,26,30,38,39], behavioral integrity [22,28,38,39], trust-building [22,23,25,27,31], inclusion [31], staff empowerment [23,35,37], collaboration [22,28,29,31,33,36], shared decision-making [22,28,34,35,37] and support for speaking up [22,23,24,25,27,29,30,31,35,41].

These behaviors help reduce fear and blame and contribute to non-retaliatory, non-punitive, and non-judgemental environments [25,29,30,31,32,34,35] in which nurses feel more comfortable voicing concerns, opinions, and ideas [24,28,31,32,34,35,38]. Nurse managers were therefore described as central agents in shaping relational and organizational conditions that allow nursing teams to communicate openly and engage in learning-oriented practices [21,25,26,27,28,29,30].

### 3.2. Role of Nurse Managers in Promoting Patient Safety Culture

The second dimension focused on the role of nurse managers in promoting patient safety culture. Nurse managers were reported to reinforce safety priorities [26,38,39,41], foster authentic and transformational leadership [20,23,31,33,39], demonstrate active listening [26,27,37], support mentoring and professional development, promote recognition [27,41], encourage nurses to advocate for patients [21,26,28], and support the reporting of adverse events, near misses, and errors [24,25,30,34,41].

The evidence also highlighted their role in promoting trust, staff involvement, empowerment, teamwork [22,23,26,27], psychologically safe and supportive work environments [22,29,30,40], and ethical and transparent practices [31]. Nurse managers were also described as important in implementing just culture principles [20,27,32], ensuring non-punitive responses to error, promoting learning rather than blame [20,21,30,32,40], supporting respectful questioning of decisions and practices [29], involving nurses in decision-making and patient safety committees [28,36,37], ensuring adequate resources and staffing [20,31,39], optimizing safety procedures and workflows [24,36,39], and supporting safety practices such as briefings, leadership rounds, SBAR communication, team training, and continuous quality improvement [21,24,28,33,39].

### 3.3. Relationship Between Psychological Safety and Patient Safety Culture

The third dimension addressed the relationship between psychological safety and patient safety culture. Psychological safety emerged as a condition, component, or facilitating mechanism that supports patient safety culture [21,26,28,29,30,31,32,38].

When nurses feel safe to speak up, question decisions, admit mistakes, report events, and participate in root cause analyses, open communication, reporting, organizational learning, and safety improvement are strengthened [20,24,25,29,30,31,32,33,34,37]. Conversely, the absence of psychological safety was associated with silence, underreporting, fear, concealment of errors, and weakening of safety culture [23,24,35,39,41,42,43].

Several included studies described psychological safety as being associated with a culture in which safety concerns can be raised, errors can be discussed constructively, and learning can occur at team and organizational levels [22,24,28,35,36,37].

### 3.4. Speaking up as a Behavioral Mechanism

The fourth dimension identified speaking up as a key behavioral mechanism linking leadership, psychological safety, and patient safety. Inclusive leadership, supervisor support, open communication, and a positive safety climate were described as factors that support nurses’ willingness to voice concerns about risks and errors [21,24,25,29,30,33,34,35].

In contrast, rigid hierarchies, fear of punishment, intimidation, bullying, abusive supervision, and lack of feedback were reported as factors that reduce nurses’ willingness to speak up [24,35,40,41,42,43]. The findings indicate that the way nurse managers respond to questions, concerns, mistakes, and reports is central to whether nurses perceive speaking up as safe and worthwhile [37,44].

Speaking up was therefore identified as a practical expression of psychological safety within nursing teams and as an important pathway through which leadership may contribute to patient safety culture [33,34].

### 3.5. Facilitators of Psychological Safety and Patient Safety Culture

The fifth dimension concerned facilitators of psychological safety and patient safety culture. These facilitators were grouped into organizational, relational, and structural or capacity-building factors.

Organizational facilitators included a just culture [27,32,42], positive safety climate [26,34], transparency [20,31], open communication [29,30,44], non-punitive responses to errors [20,30,36], organizational learning [20,28], and accessible reporting systems [20,30,32,42,44]. These factors contribute to work environments where nurses are more likely to report incidents, discuss risks, and participate in improvement processes.

Relational facilitators included inclusive, authentic, ethical, transformational, and servant leadership; visible leadership [22,23,27,28,29,31,33,35,39]; behavioral integrity [28,38]; trust [22,23,24,38]; supervisor support [30,34,35,41]; emotional and peer support [24,25,41]; interdisciplinary collaboration [28]; and constructive feedback [24,30].

Structural and capacity-building facilitators included nurses’ active participation in decision-making [26,27,28,37], empowerment [23,27], leadership and communication training [29,36,44], availability of human and material resources [20,39], adequate staffing levels [22,39], and favorable working conditions [23,40].

### 3.6. Main Barriers

The sixth dimension referred to the main barriers to psychologically safe and safety-oriented nursing environments. These included fear of blame, punishment, retaliation, humiliation, or job loss [20,25,28,38]; punitive organizational cultures [26,30,42]; low trust in management [21,28,31,33]; poor communication [26,44]; hierarchies and power differentials [23,35,37]; bullying [40]; lateral violence [22]; toxic or abusive leadership [23,31,41,43]; lack of feedback [24,33,44]; organizational silence [34,42]; high workload [29,35]; staffing shortages [29,36]; burnout [27]; post-pandemic stress [20,29]; inconsistency between policies and practice; and poorly usable reporting systems [33,44].

These barriers were described as factors that weaken psychological safety and restrict nurses’ willingness to report incidents, disclose errors, challenge unsafe decisions, or voice concerns. In particular, punitive responses to error, hierarchical work environments, and lack of feedback were repeatedly associated with silence, underreporting, and reduced learning from adverse events [24,42,43].

Some included studies suggested that nurse managers may influence these barriers both directly, through their leadership behaviors, and indirectly, through their role in shaping team climate, communication processes, and access to resources [39,44].

### 3.7. Knowledge Gaps and Future Research Needs

The seventh dimension concerned knowledge gaps and future research needs. The available evidence remains predominantly observational, relying mainly on cross-sectional, qualitative, narrative, and conceptual studies [26,27,28,29,30,31,32,33,34,35,36,37,41,42,44]. This limits understanding of causal relationships between leadership, psychological safety, speaking up, and patient safety outcomes.

The studies highlighted the need for greater conceptual and methodological standardization, particularly in the definition and measurement of psychological safety and related constructs. Longitudinal and intervention studies are also needed to assess the effectiveness of different leadership styles and organizational strategies in promoting speaking up and patient safety culture.

Further research is required on toxic and abusive leadership [39,41,43], organizational silence [42], discrepancies between nurse managers’ and nurses’ perceptions [26], and the influence of different organizational and cultural contexts on the transferability of findings [36].

### 3.8. Integrated Conceptual Framework

**Figure 2:**
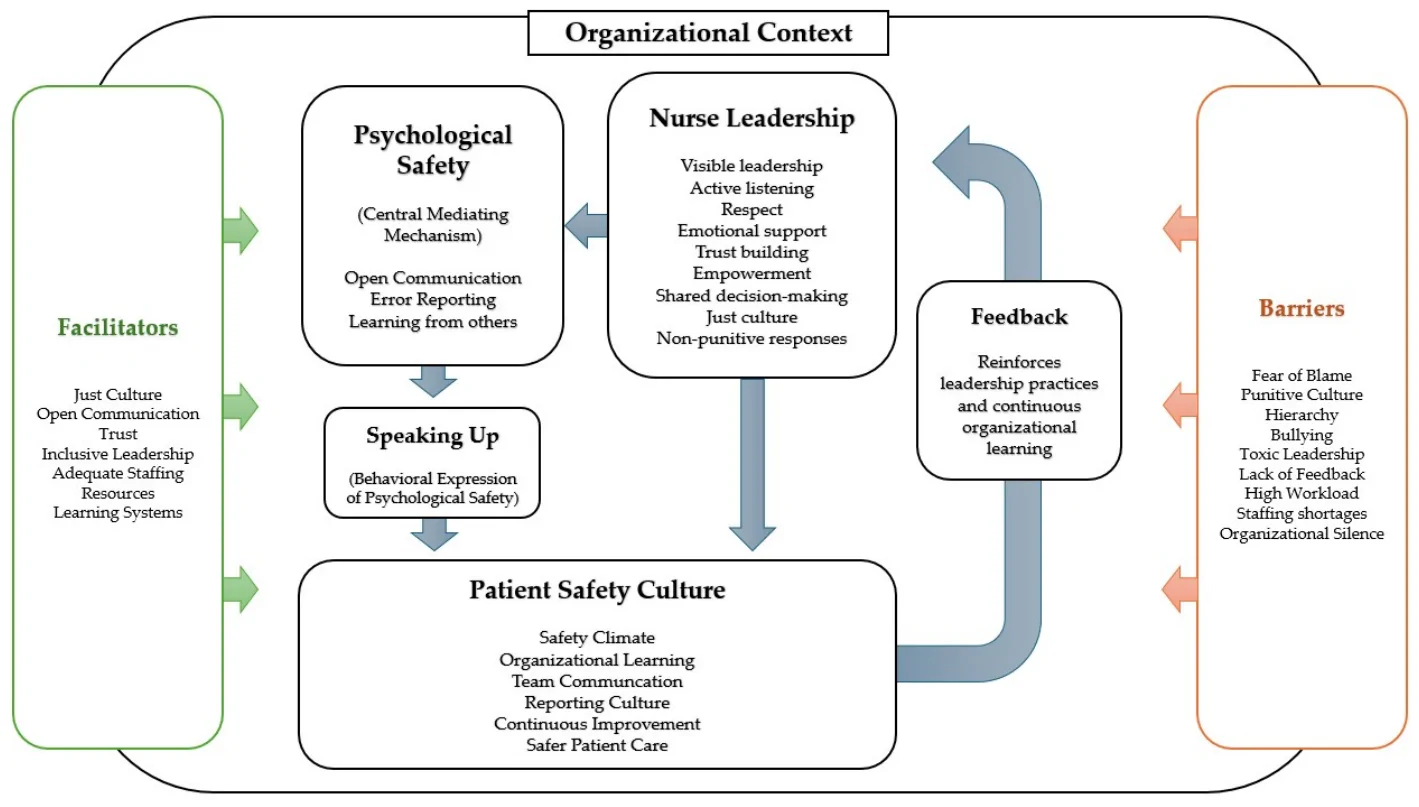
Conceptual model developed through thematic synthesis of included studies (authors’ interpretation of reviewed evidence).

Based on the thematic synthesis, an integrated conceptual framework was developed to illustrate the relationships identified across the included studies (Figure 2). It should be noted that this conceptual model represents the authors’ synthesis and interpretation of the included evidence through thematic analysis. As such, it is intended as a conceptual framework to support understanding of the phenomenon rather than as an empirically validated model. The framework positions nurse manager leadership as a key influence on psychological safety, which appears to be a key element linking leadership practices to speaking up behaviors and patient safety culture. It also highlights the influence of facilitators, barriers, organizational context, and feedback processes in shaping these relationships.

## 4. Discussion

This scoping review identifies psychological safety as a central organizational mechanism underpinning patient safety culture and highlights nurse managers as key actors in its development. While this finding aligns with the broader healthcare leadership literature [3,12,45], the present synthesis advances current knowledge by conceptualizing psychological safety not merely as an outcome of leadership, but as an intermediate mechanism through which leadership practices translate into safety-related behaviors and organizational learning.

One of the most consistent findings across the included studies is the central role of nurse managers’ relational and communicative behaviors in shaping psychologically safe environments [20,21,22,23,24,25,26,27,28,29,30,31,32,33,34,35,36,37,38,39,41,42]. Across these studies, leadership practices such as visible presence, active listening, respectful engagement, emotional support, and behavioral integrity were repeatedly associated with higher levels of trust and psychological safety, contributing to a reduced perception of interpersonal risk among nursing staff. These findings are consistent with existing literature, which highlights the role of relational leadership behaviors in fostering trust and minimizing fear of negative interpersonal consequences in healthcare teams [39,46]. In line with established theoretical frameworks of psychological safety, such environments appear to enable nurses to engage in interpersonal risk-taking behaviors—including questioning clinical decisions, admitting errors, and voicing concerns—which are essential for speaking up and maintaining patient safety [1,26].

The findings also suggest a notable convergence across different leadership approaches identified in the included studies, including transformational, inclusive, ethical, authentic, and servant leadership [23,30,31,33,35,39]. While these frameworks are theoretically distinct, they appear to share a common set of relational attributes—such as trust-building, empowerment, open communication, and staff engagement—that are consistently linked to both psychological safety and patient safety culture within the reviewed evidence. This convergence suggests that the effectiveness of leadership in fostering psychologically safe environments may be less dependent on adherence to a specific theoretical model and more on the consistent enactment of relationally grounded leadership behaviors. Such an interpretation is supported by emerging evidence emphasizing the practical importance of everyday leadership practices over formal leadership typologies in shaping team dynamics and safety outcomes [47].

Importantly, this review supports the proposition that psychological safety may function as a mediating pathway linking leadership practices to patient safety–related processes and outcomes [20,27,28,30,32,36].

The included studies consistently suggest that when nurse managers foster environments in which nurses feel safe to speak up [24,29], communication processes are strengthened, incident reporting is more likely to occur, and opportunities for learning from errors are enhanced [34]. These mechanisms are widely recognized as essential to the functioning of high-reliability organizations, where early identification of risks and responsiveness to weak signals are critical to preventing harm [48,49]. Conversely, the absence of psychological safety appears to contribute to silence, underreporting, and missed opportunities for organizational learning [40,41,42], ultimately undermining the development of a robust patient safety culture [3,50]. In light of these findings, several implications for nursing practice can be identified. The centrality of relational leadership behaviors highlighted in this review suggests that the development of nurse managers should prioritize competencies such as active listening, respectful engagement, emotional support, and the ability to foster open and trusting team environments [39,46]. Leadership development interventions that emphasize these relational dimensions—alongside visible and accessible leadership practices, such as leader rounding—may play an important role in strengthening psychological safety in clinical settings [46]. At an organizational level, recognizing psychological safety as a modifiable determinant of patient safety supports its integration into quality improvement and safety monitoring systems [3]. In addition, the implementation of structured strategies to support speaking up—including non-punitive reporting systems, briefings and debriefings, and team reflexivity practices—may enhance communication, facilitate learning from errors, and ultimately improve patient safety outcomes [1,26]. Collectively, these implications reinforce the importance of embedding relationally grounded leadership practices in nursing management as a means of advancing both safety culture and quality of care.

Within this framework, speaking up emerges as a key behavioral expression of psychological safety [21,24,25,26,29,35]. The findings indicate that nurses’ willingness to raise concerns is highly sensitive to leadership responses. Supportive, non-punitive, and constructive reactions appear to reinforce speaking up as a valued and safe practice, whereas punitive or dismissive responses contribute to fear, silence, and withdrawal. This reinforces the view that psychological safety is not only enabled by leadership but continuously negotiated through dynamic feedback loops between staff behaviors and managerial responses [51].

The analysis of facilitators and barriers further underscores that psychological safety and patient safety culture are shaped by multilevel factors. The conceptual framework developed from this review further illustrates how leadership competencies, psychological safety, speaking up behaviors, organizational conditions, and patient safety culture interact as part of a dynamic and interdependent system rather than as isolated determinants. While relational leadership behaviors are essential [26,27,33,34,36], they appear insufficient in isolation. Organizational conditions—such as just culture, transparent processes, accessible reporting systems, and alignment between policy and practice—play a critical role in enabling or constraining leadership effectiveness [32,44]. The adoption of a just culture is particularly important, as fear of blame and punishment remains a major barrier to reporting adverse events and near misses, while also being positively associated with patient safety activities among nurses [52,53].

Similarly, structural conditions, including adequate staffing, resource availability, and workload, influence whether nurses have the practical capacity to engage in communication, reporting, and learning activities. Resource constraints [30] and high workload have been shown to undermine psychological safety and contribute to burnout, particularly in high-pressure healthcare contexts [27]. The findings also suggest that organizational barriers may alter how leadership competencies are enacted in practice. Feedback provides a useful example. While constructive and timely feedback is consistently identified as a key leadership behavior supporting psychological safety [26,30,35,44], excessive workload and staffing pressures may restrict managers’ availability to observe practice, engage in dialog with staff, and provide follow-up after incident reporting [9,24,27,34]. In this way, workload does not simply reduce the amount of feedback delivered; it may transform feedback from a developmental and relationship-building process into a task-focused or sporadic activity, thereby reducing its contribution to trust, learning, and psychological safety [26,30,35,44]. These findings reinforce a systemic perspective on patient safety, whereby outcomes emerge from the interaction between individual, relational, and organizational domains [4,8,48,53].

At the same time, the review highlights the persistence of significant barriers, including punitive cultures, hierarchical structures, bullying and toxic leadership, lack of feedback, and high workload [21,28,29,35,39,40]. These factors not only undermine psychological safety but also contribute to organizational silence and disengagement. Notably, several of these barriers extend beyond the direct control of nurse managers, pointing to the importance of alignment between frontline leadership, senior management, and institutional policies. This suggests that efforts to strengthen psychological safety and patient safety culture must be supported by organization-wide strategies, rather than relying solely on individual leadership competencies [53].

The findings also highlight an important tension between individual leadership agency and institutional constraints. While relational leadership behaviors are consistently associated with psychological safety, their capacity to generate sustainable change may be limited in organizational cultures characterized by rigid hierarchies, power asymmetries, and punitive responses to error [23,37,38,39,41,51]. In such environments, nurse managers may support open communication and speaking up within their immediate teams, yet broader organizational norms and structures can undermine these efforts by reinforcing fear, silence, and deference to authority [21,23,32,38,39,41]. This suggests that psychological safety should not be viewed solely as the product of individual leadership competencies, but rather as an emergent property of interactions between leadership practices, organizational culture, and institutional structures [1,42,43,48]. Consequently, relying exclusively on nurse managers to foster psychological safety may place unrealistic expectations on frontline leaders when wider organizational conditions remain unchanged.

From a practice perspective, this review suggests that leadership development programs for nurse managers should prioritize relational, ethical, and communication competencies, including feedback practices, conflict management, and strategies to support speaking up [29,30,33,36,37,40]. However, such initiatives are unlikely to be effective without concurrent organizational interventions that ensure non-punitive reporting systems, adequate staffing, and consistent implementation of just culture principles. Fear of blame remains a major barrier to reporting, highlighting the importance of just culture approaches that promote learning and psychological safety [53]. Patient safety culture is influenced not only by leadership behaviors but also by broader organizational conditions, including staffing, reporting systems, and institutional support [39,50]. Strengthening safety culture therefore requires coordinated, multilevel interventions rather than isolated leadership initiatives [7]. The findings also have implications for healthcare executives and policy makers. Beyond supporting leadership development, organizations may benefit from incorporating psychological safety indicators into management evaluation and quality-monitoring systems. Examples include staff perceptions of communication openness, willingness to speak up, psychological safety scores, incident reporting climate, and feedback quality [23,26,30,43]. Such indicators may provide a more comprehensive assessment of leadership effectiveness than traditional operational metrics alone. Furthermore, leadership development programs should move beyond theoretical training and prioritize practical competencies identified in this review, including active listening, constructive feedback, conflict management, facilitation of difficult conversations, just culture implementation, and strategies for responding constructively to staff concerns and incident reports [9,29,36,44]. Embedding these competencies within leadership standards, performance appraisal systems, and continuing professional development programs may strengthen the sustainability of psychological safety and patient safety culture initiatives.

Future research should prioritize longitudinal and intervention studies to better understand causal pathways and to assess the effectiveness of leadership and organizational strategies in promoting psychological safety and patient safety outcomes. Further investigation is also needed into underexplored areas, including toxic and abusive leadership, organizational silence, perceptual gaps between nurse managers and staff nurses, and the influence of cultural and organizational contexts on the transferability of findings. Comparative studies across healthcare systems and cultural settings would be particularly valuable for identifying context-specific and context-independent factors that influence the relationship between nurse manager leadership, psychological safety, and patient safety culture. Overall, this scoping review highlights the critical role of nurse managers in promoting psychological safety and patient safety culture within nursing teams. By synthesizing evidence across diverse studies, it demonstrates how relational leadership behaviors shape psychologically safe environments that enable communication, speaking up, and learning from errors. Importantly, the findings suggest that psychological safety may function as a potential mediating mechanism through which nurse managers’ leadership practices influence safety-related behaviors and the development of patient safety culture. While nurse managers play a central role in this process, their impact appears contingent upon supportive organizational conditions, including just culture, adequate staffing, and effective systems for reporting and feedback. These findings underscore the need for coordinated, multilevel strategies that align leadership practices with organizational structures. Taken together, this review advances current understanding by offering an integrated perspective on how nurse management, psychological safety, and organizational context interact to support safer care delivery in nursing teams.

### Strengths and Limitations of Review

This review has several strengths. It addresses a timely and clinically relevant topic, given the increasing recognition of psychological safety and patient safety culture as key determinants of high-quality nursing care and patient outcomes. To our knowledge, this is the first scoping review to comprehensively map the evidence on the role of nurse managers in promoting psychological safety and patient safety culture. The inclusion of diverse evidence sources, including empirical studies, conceptual papers, expert opinion papers, and gray literature, enabled a broad understanding of this emerging field. Furthermore, the review was conducted in accordance with the JBI methodology and reported following the PRISMA-ScR guidelines, enhancing methodological transparency and reproducibility.

Despite these strengths, the findings should be interpreted in light of certain methodological limitations in the existing evidence base. The predominance of cross-sectional and qualitative designs not only limits the ability to establish causal relationships between leadership, psychological safety, and patient safety outcomes, but also restricts understanding of the temporal dynamics through which these constructs may influence one another. Additionally, conceptual and measurement heterogeneity—particularly regarding psychological safety, safety culture, and related constructs—restricts comparability across studies and poses challenges for evidence synthesis. These limitations highlight the need for greater conceptual clarity and more rigorous research designs [12,54].

A further limitation relates to the geographical and contextual diversity of the included studies. Although the evidence was generated across different countries, most studies originated from the United States and South Korea, with relatively limited evidence from other healthcare systems. Furthermore, the included studies were conducted across diverse healthcare organizations, and nursing practice environments, each characterized by distinct cultural norms, leadership expectations, regulatory frameworks, and patient safety infrastructures. These differences may influence how psychological safety is understood, enacted, and measured, as well as how leadership behaviors are perceived and responded to by nursing staff. For example, the willingness to speak up may be shaped by national and organizational cultures regarding hierarchy, authority, and communication, while the implementation of patient safety initiatives may depend on the resources, staffing models, and reporting systems available within specific healthcare systems. Consequently, caution is warranted when generalizing the findings across settings, as leadership practices shown to support psychological safety in one context may not be equally effective or feasible in another. Future research conducted across a broader range of countries and healthcare systems would strengthen the international applicability of the evidence.

Consistent with scoping review methodology, no formal methodological quality appraisal of the included studies was undertaken. Consequently, the findings should not be interpreted as reflecting the strength or certainty of the available evidence, nor should they be used to support evidence-based recommendations.

## 5. Conclusions

This scoping review highlights the central role of nurse managers in fostering psychological safety and strengthening patient safety culture within nursing teams. The findings suggest that psychological safety may function as an important mechanism through which leadership practices influence communication, speaking up, organizational learning, and safety-related behaviors.

Although nurse managers are well positioned to promote psychologically safe and safety-oriented work environments, the enactment and effectiveness of leadership competencies depend on supportive organizational conditions, including a just culture, adequate resources, and systems that encourage learning and open communication.

By providing an integrated synthesis of evidence on psychological safety, patient safety culture, and nurse manager leadership, this review contributes to a more comprehensive understanding of the factors that support safer and higher-quality nursing care. The findings offer useful insights for leadership development, organizational decision-making, and initiatives aimed at strengthening safety culture across healthcare settings.

However, further longitudinal and intervention studies are needed to determine the effectiveness of specific leadership approaches in promoting psychological safety, patient safety culture, and related outcomes.

## Figures and Tables

**Table 1:** Eligibility criteria and definitions.

Category	Criterion	Definition
Population	Nurse Administrators	Nurses professionally qualified in administration [18].
Concept	Psychological Safety	An environment where people are able to act and engage without fear of punishment or humiliation from others [18].
	Patient Safety Culture	Shared values, attitudes, competencies, and behavioral patterns that determine an organization’s commitment to safety and its ability to manage safety effectively [4,5].
Context	Healthcare Settings	Any healthcare setting, regardless of country of origin or sociocultural environment.

**Table 2:** Thematic synthesis of the evidence: key findings across included studies.

Dimension	Key Findings	Supporting Studies
Role of nurse managers in promoting psychological safety	Visible leadership; active listening; emotional support; behavioral integrity; trust-building; equity and inclusion; open communication; staff empowerment; collaboration; shared decision-making; support for speaking up; creation of non-punitive and non-retaliatory environments.	[20,21,22,23,24,25,26,27,28,29,30,31,32,33,34,35,36,37,38,39]
Role of nurse managers in promoting patient safety culture	Authentic and transformational leadership; mentoring; professional development; staff recognition; teamwork; communication openness; support for incident reporting and learning; implementation of just culture principles; nurse involvement in decision-making and safety committees; staffing and resource management; safety briefings, leadership rounds, SBAR, team training, and quality improvement initiatives.	[20,21,22,23,24,25,26,27,28,29,30,31,32,33,34,36,37,38,39,40,41]
Relationship between psychological safety and patient safety culture	Psychological safety supports communication openness, incident reporting, organizational learning, and safety improvement. Lack of psychological safety is associated with silence, fear of reporting, and weakened safety culture.	[20,21,22,23,24,25,26,28,29,30,31,32,34,35,36,37,38,39,41,42,43]
Speaking up as a behavioral mechanism	Voice behaviors; raising concerns; questioning decisions and practices; reporting incidents and errors; knowledge sharing; participation in root cause analysis; organizational learning; safety improvement.	[21,24,25,26,29,30,33,34,35,37,40,41,42,44]
Facilitators	**Organizational**: just culture, transparency, reporting systems, organizational learning. **Relational**: inclusive/authentic/transformational leadership, trust, support, collaboration, feedback. **Structural:** empowerment, participation in decision-making, training, adequate staffing and resources.	[20,22,23,24,25,26,27,28,29,30,31,32,33,34,35,36,37,38,39,40,41,42,44]
Main barriers	Fear of blame, punishment, retaliation, humiliation; punitive cultures; low trust in management; poor communication; hierarchy; bullying; toxic leadership; organizational silence; workload; staffing shortages; burnout; limited feedback; poor reporting systems.	[20,21,22,23,24,25,26,27,28,29,30,31,33,35,36,37,38,39,40,41,42,43,44]
Knowledge gaps and future research needs	Limited causal evidence; predominance of cross-sectional and qualitative studies; need for conceptual standardization; need for longitudinal and intervention studies; further research on toxic leadership, organizational silence, perceptual discrepancies, and contextual influences.	[26,27,28,36,39,41,42,43,44]

## Data Availability

The OSF registration [16] contains the study protocol only. The extraction form, coded dataset, excluded full-text list, and other review materials are not publicly available through the OSF repository. These materials are available from the corresponding author upon reasonable request.

## Supplementary Materials

The following supporting information can be downloaded at: https://www.mdpi.com/article/10.3390/healthcare14162590/s1, Table S1. Complete search strategies used for each electronic database. Table S2. Summary of included studies according to study characteristics and extracted thematic information. Preferred Reporting Items for Systematic reviews and Meta-Analyses extension for Scoping Reviews (PRISMA-ScR) Checklist.
