# Supplementary material for: The Role of Nurse Managers in Promoting Psychological Safety and Patient Safety Culture in Nursing Teams: A Scoping Review
Source: Healthcare (Basel). 2026 Aug 18;14(16):2590. doi: 10.3390/healthcare14162590 (PMC13512081; doi:10.3390/healthcare14162590)
Supplement: Supplementary file 1 [file healthcare-14-02590-s001.zip › Supplementary material_Table S1.pdf]

Supplementary Table S1. Complete search strategies used for each electronic database.

Database: PubMed (MEDLINE)

Search date: May 11, 2026

Search	Query	Records retrieved
#1	"Nurse Administrators"[MeSH Terms] OR "Nurse Administrator*"[Title/Abstract] OR “nurse executive*”[Title/Abstract] OR "nurse manager*"[Title/Abstract] OR “nurse leader*”[Title/Abstract] OR “nursing leadership”[Title/Abstract] OR “Head Nurse*”[Title/Abstract] OR “ward manager*”[Title/Abstract]	23,689
#2	"Psychological Safety”[MeSH Terms] OR “psychological safety”[Title/Abstract] OR “psychological saf*”[Title/Abstract] OR “team psychological safety”[Title/Abstract] OR “workplace psychological safety”[Title/Abstract] OR “psychosocial safety”[Title/Abstract] OR “communication openness”[Title/Abstract] OR “organizational trust”[Title/Abstract] OR “speaking up”[Title/Abstract] OR “team climate”[Title/Abstract] OR “interpersonal safety”[Title/Abstract]	3,294
#3	"patient safety"[MeSH Terms] OR "patient safet*"[Title/Abstract] OR "patient harm"[MeSH Terms] OR "patient harm"[Title/Abstract] OR "safety management"[MeSH Terms] OR "patient risk*"[Title/Abstract] OR "safety"[MeSH Terms] OR "safety culture"[Title/Abstract] OR "safety climate"[Title/Abstract] OR "patient safety culture"[Title/Abstract] OR "medical errors"[MeSH Terms] OR "adverse event"[Title/Abstract] OR "patient outcomes"[Title/Abstract]	398,807
#4	("Nurse Administrators"[MeSH Terms] OR "nurse administrator*"[Title/Abstract] OR "nurse executive*"[Title/Abstract] OR "nurse manager*"[Title/Abstract] OR "nurse leader*"[Title/Abstract] OR "nursing leadership"[Title/Abstract] OR	38

	"head nurse*"[Title/Abstract] OR "ward manager*"[Title/Abstract]) AND ("Psychological Safety"[MeSH Terms] OR "Psychological Safety"[Title/Abstract] OR "psychological saf*"[Title/Abstract] OR "team psychological safety"[Title/Abstract] OR "workplace psychological safety"[Title/Abstract] OR "psychosocial safety"[Title/Abstract] OR "communication openness"[Title/Abstract] OR "organizational trust"[Title/Abstract] OR "speaking up"[Title/Abstract] OR "team climate"[Title/Abstract] OR "interpersonal safety"[Title/Abstract]) AND ("patient safety"[MeSH Terms] OR "patient safet*"[Title/Abstract] OR "patient harm"[MeSH Terms] OR "patient harm"[Title/Abstract] OR "safety management"[MeSH Terms] OR "patient risk*"[Title/Abstract] OR "safety"[MeSH Terms] OR "safety culture"[Title/Abstract] OR "safety climate"[Title/Abstract] OR "patient safety culture"[Title/Abstract] OR "medical errors"[MeSH Terms] OR "adverse event"[Title/Abstract] OR "patient outcomes"[Title/Abstract])	

Database: CINAHL Ultimate (EBSCOhost)

Search date: May 11, 2026

Search	Query	Records retrieved
#1	XB (MH "Nurse Administrators" OR "Nurse Administrator*" OR "nurse executive*" OR "nurse manager*" OR "nurse leader*" OR "nursing leadership" OR "Head Nurse*" OR "ward manager*")	23,153
#2	XB (MH "Psychological Safety" OR "psychological saf*" OR "team psychological safety" OR "workplace psychological safety" OR "psychosocial safety" OR "communication openness" OR "organizational trust" OR "speaking up" OR "team climate" OR "interpersonal safety")	2,456
#3	XB (MH "Patient Safety" OR MH "Health Care Errors" OR MH "Safety" OR "patient safet*" OR "patient harm" OR "safety management" OR "patient risk*" OR "safety" OR "safety culture" OR "safety climate" OR "patient safety culture" OR "medical errors" OR "adverse event" OR "patient outcomes")	369,681
#4	(XB (MH "Nurse Administrators" OR "Nurse Administrator*" OR "nurse executive*" OR "nurse manager*" OR "nurse leader*" OR "nursing leadership" OR "Head Nurse*" OR "ward manager*")) AND (XB (MH "Psychological Safety" OR "psychological saf*" OR "team psychological safety" OR "workplace psychological safety" OR "psychosocial safety" OR "communication openness" OR "organizational trust" OR "speaking up" OR "team climate" OR "interpersonal safety")) AND (XB (MH "Patient Safety" OR MH "Health Care Errors" OR MH "Safety" OR "patient safet*" OR "patient harm" OR "safety management" OR "patient risk*" OR "safety" OR "safety culture" OR "safety climate" OR "patient safety culture" OR "medical errors" OR "adverse event" OR "patient outcomes"))	78

Database: Psychology and Behavioral Sciences Collection (EBSCOhost)

Search date: May 11, 2026

Search	Query	Records retrieved
#1	XB (DE "Nurse administrators" OR "Nurse Administrator*" OR “nurse executive*” OR "nurse manager*" OR “nurse leader*” OR “nursing leadership” OR “Head Nurse*” OR “ward manager*”)	232
#2	XB (DE "Psychological safety" OR DE "Risk-taking behavior" OR DE "Harm reduction" OR “psychological saf*” OR “team psychological safety” OR “workplace psychological safety” OR “psychosocial safety” OR “communication openness” OR “organizational trust” OR “speaking up” OR “team climate” OR “interpersonal safety”)	5,708
#3	XB (DE "Patient Safety" OR MH "Health Care Errors" OR MH "Safety" OR "patient safet*" OR "patient harm" OR "safety management" OR "patient risk*" OR "safety" OR "safety culture" OR "safety climate" OR "patient safety culture" OR "medical errors" OR "adverse event" OR "patient outcomes")	27,149
#4	(XB (DE "Nurse administrators" OR "Nurse Administrator*" OR “nurse executive*” OR "nurse manager*" OR “nurse leader*” OR “nursing leadership” OR “Head Nurse*” OR “ward manager*”)) AND (XB (DE "Psychological safety" OR DE "Risk-taking behavior" OR DE "Harm reduction" OR “psychological saf*” OR “team psychological safety” OR “workplace psychological safety” OR “psychosocial safety” OR “communication openness” OR “organizational trust” OR “speaking up” OR “team climate” OR “interpersonal safety”)) AND (XB (DE "Patient Safety" OR MH "Health Care Errors" OR MH "Safety" OR "patient safet*" OR "patient harm" OR "safety management" OR "patient risk*" OR "safety" OR "safety culture" OR "safety climate" OR "patient safety culture" OR "medical errors" OR "adverse event" OR "patient outcomes"))	3

Database: Scopus

Search date: May 11, 2026

Search	Query	Records retrieved
#1	ABS ("Nurse Administrators" OR "Nurse Administrator*" OR "nurse executive*" OR "nurse manager*" OR "nurse leader*" OR "nursing leadership" OR "Head Nurse*" OR "ward manager*")	13,430
#2	ABS ("Psychological Safety" OR "psychological safety" OR "psychological saf*" OR "team psychological safety" OR "workplace psychological safety" OR "psychosocial safety" OR "communication openness" OR "organizational trust" OR "speaking up" OR "team climate" OR "interpersonal safety")	8,003
#3	ABS("patient safety" OR "patient safet*" OR "patient harm" OR "patient harm" OR "safety management" OR "patient risk*" OR "safety" OR "safety culture" OR "safety climate" OR "patient safety culture" OR "medical errors" OR "adverse event" OR "patient outcomes")	2,246,477
#4	ABS ("patient safety" OR "patient safet*" OR "patient harm" OR "patient harm" OR "safety management" OR "patient risk*" OR "safety" OR "safety culture" OR "safety climate" OR "patient safety culture" OR "medical errors" OR "adverse event" OR "patient outcomes") AND ABS ("Nurse Administrators" OR "Nurse Administrator*" OR "nurse executive*" OR "nurse manager*" OR "nurse leader*" OR "nursing leadership" OR "Head Nurse*" OR "ward manager*") AND ABS ("Psychological Safety" OR "psychological safety" OR "psychological saf*" OR "team psychological safety" OR "workplace psychological safety" OR "psychosocial safety" OR "communication openness" OR "organizational trust" OR "speaking up" OR "team climate" OR "interpersonal safety")	90

Database: BVS

Search date: May 11, 2026

(ab:(("Nurse Administrators" OR "Nurse Administrator*" OR "nurse executive*" OR "nurse manager*" OR "nurse leader*" OR "nursing leadership" OR "Head Nurse*" OR "ward manager*"))) AND (ab:(("Psychological Safety" OR "psychological safety" OR "psychological saf*" OR "team psychological safety" OR "workplace psychological safety" OR "psychosocial safety" OR "communication openness" OR "organizational trust" OR "speaking up" OR "team climate" OR "interpersonal safety"))) AND (ab:(("patient safety" OR "patient safet*" OR "patient harm" OR "patient harm" OR "safety management" OR "patient risk*" OR "safety" OR "safety culture" OR "safety climate" OR "patient safety culture" OR "medical errors" OR "adverse event" OR "patient outcomes")))

Database: Worldcat

Search date: May 11, 2026

“patient safety” AND “psychological safety” AND “nurse”

Filters: “Thesis, dissertation”
