# Supplementary material for: The Role of Nurse Managers in Promoting Psychological Safety and Patient Safety Culture in Nursing Teams: A Scoping Review
Source: Healthcare (Basel). 2026 Aug 18;14(16):2590. doi: 10.3390/healthcare14162590 (PMC13512081; doi:10.3390/healthcare14162590)
Supplement: Supplementary file 1 [file healthcare-14-02590-s001.zip › Supplementary material_TableS2.pdf]

Table S2

Summary of included studies according to study characteristics and extracted thematic information

Title<br>Author(s)<br>(Year)	Country of origin	Type of study	Goal(s)	Context	Nurse managers and psychological safety	Nurse managers and patient safety culture	Relationship between psychological safety and patient safety culture	Facilitators and barriers
Answering the Wake-Up Call to Nurse Leaders: Five Practices to Restore Psychological Safety After the Vaught Case<br><br>Camilleri et al. (2023)	USA	Expert opinion article	To propose leadership practices for nurse leaders to restore psychological safety, trust, and patient safety culture following the Vaught case.	Post-pandemic healthcare environment following the RaDonda Vaught criminal prosecution; nursing workforce experiencing stress, fear, reduced trust, and threats to psychological safety and patient safety culture.	✓	✓	✓	✓
A Likert-Type scale for evaluating the "bottom line" of patient safety<br><br>Sarit Rashkovits (2020)	Israel	Scale development and validation / quantitative cross-sectional	To develop a scale evaluating the perceived state of "almost no adverse events" and test its relationship with leadership, psychological safety, and safety behavior.	Various healthcare organizations and wards	✓	✓	✓	✓
The Role of Authentic Leadership in Nursing and Healthcare Wong<br><br>Carol Wong (2008)	Canada	PhD Thesis comprising a systematic review, a conceptual paper, and quantitative empirical studies.	To examine the influence of authentic leadership on nurses' and healthcare providers' work outcomes and its contribution to healthy work environments and patient safety.	Healthcare settings including acute care hospital settings and nursing practice environments.	✓	✓	✓	✓

Table S2

Summary of included studies according to study characteristics and extracted thematic information

Title Author(s) (Year)	Country of origin	Type of study	Goal(s)	Context	Nurse managers and psychological safety	Nurse managers and patient safety culture	Relationship between psychological safety and patient safety culture	Facilitators and barriers
Describing Nurse Leaders' and Direct Care Nurses' Perceptions of a Healthy Work Environment in Acute Care Settings, Part 2<br><br>Huddleston & Gray (2016)	USA	Qualitative descriptive study	To explore nurse leaders' and direct care nurses' perceptions of the meaning of a healthy work environment (HWE), describe these perceptions, and identify the characteristics of a HWE in acute care settings.	Acute care hospital settings	✓	✓	✓	✓
Demystifying the Experience of Participating in a Root Cause Analysis: A Hermeneutic Phenomenological Study<br><br>Abreu et al. (2023)	USA	Qualitative study – Hermeneutic phenomenological design	To explore registered nurses' experiences of participating in a Root Cause Analysis (RCA) meeting following their involvement in an adverse event.	Healthcare settings in the United States, including hospitals, long-term care facilities, rehabilitation facilities, ambulatory care clinics, and other care settings where RCA investigations are conducted following adverse events.	✓	✓	✓	✓
Speaking Up Behaviours (Safety Voices) of Healthcare Workers: A Metasynthesis of Qualitative Research Studies	USA	Interpretive metasynthesis of qualitative studies (qualitative evidence synthesis)	To develop an understanding of how nurses and other healthcare workers relate to safety voice (speaking-up behaviours) and how these behaviours influence clinical practice and patient safety.	Multiple healthcare settings internationally, including hospitals, operating rooms, oncology units, perioperative settings, and acute care environments. The metasynthesis included 11 qualitative studies	✓	✓	✓	✓

Table S2

Summary of included studies according to study characteristics and extracted thematic information

Title<br>Author(s)<br>(Year)	Country of origin	Type of study	Goal(s)	Context	Nurse managers and psychological safety	Nurse managers and patient safety culture	Relationship between psychological safety and patient safety culture	Facilitators and barriers
Morrow et al. (2016)				involving 504 healthcare professionals, including 354 nurses.				
Speaking up about patient safety concerns: the influence of safety management approaches and climate on nurses' willingness to speak up<br><br>Carien W Alingh; Jeroen D H van Wijngaarden; Karina van de Voorde; Jaap Paauwe; Robbert Huijsman (2018)	Netherlands	Quantitative cross-sectional study	To explore relationships between control-based and commitment-based safety management, climate for safety, psychological safety, and nurses' speaking-up attitudes.	Hospital setting; 93 clinical wards including medical, surgical, mixed medical/surgical wards, and intensive care units (ICUs) involving nurse managers and nursing teams	✓	✓	✓	✓
Engaging the Nursing Workforce: The KINDER Framework for Nurse Leaders<br><br>Reynaldo R. Rivera & Alexandra N. Shelley (2025)	USA	Conceptual / evidence-informed framework paper	To present the KINDER framework (Know, Involve, Nurture, Drive, Empower, Recognize) as an evidence-based strategy to enhance nurse engagement and support nurse leaders in developing resilient and empowered nursing teams.	Healthcare organizations and nursing workforce settings; intended for implementation across clinical nursing environments and leadership development programmes	✓	✓	✓	✓

Table S2

Summary of included studies according to study characteristics and extracted thematic information

Title<br>Author(s)<br>(Year)	Country of origin	Type of study	Goal(s)	Context	Nurse managers and psychological safety	Nurse managers and patient safety culture	Relationship between psychological safety and patient safety culture	Facilitators and barriers
Safety culture in health care teams: A narrative review of the literature<br><br>Roisin O'Donovan, Marie Ward, Aoife De Brún, Eilish McAuliffe (2019)	Ireland	Narrative review	To explore the role of nurse managers in promoting psychological safety and patient safety culture in nursing teams; specifically to examine factors affecting safety culture within healthcare teams.	Healthcare organisations across multiple settings (hospitals, primary care, surgical units, intensive care units)<br><br>Publications from 2006–2017<br><br>International scope (studies from various countries, reviewed from major databases)	✓	✓	✓	✓
Influence of Workplace Bullying and Bystander Types on Speaking Up for Patient Safety Among Hospital Nurses: A Cross-Sectional Study<br><br>Park et al. (2025)	South Korea	Cross-sectional study	To explore the influences of workplace bullying experiences, witnessing workplace bullying and bystander types on Speaking up for patient safety (SUPS) among hospital nurses.	Acute hospital care setting (tertiary hospitals) in South Korea, involving nursing teams working in inpatient wards and ICUs.	✓	✓	✓	✓
Psychological safety, communication openness, nurse job outcomes, and patient safety in hospital nurses	USA	Cross-sectional study	To examine the relationships between psychological safety at work and job outcomes (i.e., job satisfaction and intention to leave), and patient	US hospital inpatient nursing settings during the COVID-19 pandemic.	✓	✓	✓	✓

Table S2

Summary of included studies according to study characteristics and extracted thematic information

Title<br>Author(s)<br>(Year)	Country of origin	Type of study	Goal(s)	Context	Nurse managers and psychological safety	Nurse managers and patient safety culture	Relationship between psychological safety and patient safety culture	Facilitators and barriers
Cho et al. (2023)			safety among hospital nurses; and to determine the mediating effect of communication openness on these relationships.					
Hospital management, supervisor support and nurse speakingup behaviours: The mediating role of safety culture perception<br><br>Seo et al. (2022)	South Korea	Cross-sectional correlational study (secondary analysis of survey data)	To (a) elucidate the effects of hospital management and unit supervisor support for patient safety on nurse speaking-up behaviours and<br>(b) identify potential mediators of this relationship.	Acute care teaching hospital in South Korea; inpatient nursing practice context focused on patient safety culture and speaking-up behaviours	✓	✓	✓	✓
Authentic Leadership, Psychological Safety, Missed Nursing Care, and Intention to Leave Among Hospital Nurses<br><br>Cho et al. (2025)	USA	Cross-sectional descriptive study	To examine the relationships between authentic leadership, missed nursing care, and intention to leave in hospital nurses and to investigate the mediating role of psychological safety in these relationships	Acute care hospital setting in the United States; inpatient nursing units focused on authentic leadership, psychological safety, missed nursing care, and nurse retention.	✓	✓	✓	✓
The influence of organizational culture, climate and commitment on speaking up	USA	Quantitative cross-sectional survey study	To understand how a hospital's organisational culture, climate, and commitment influence nurses' and healthcare workers' intentions to	A single mid-size Midwestern US hospital; 162 medical professionals and support staff including	✓	✓	✓	✓

Table S2

Summary of included studies according to study characteristics and extracted thematic information

Title<br>Author(s)<br>(Year)	Country of origin	Type of study	Goal(s)	Context	Nurse managers and psychological safety	Nurse managers and patient safety culture	Relationship between psychological safety and patient safety culture	Facilitators and barriers
about medical errors<br><br>Kenneth J. Levine; Molly Carmody; Kami J. Silk<br>(2020)			speak up about medical errors	nurses, physicians, and other caregivers				
Hospital Nurse Leaders Perceptions of Just Culture<br><br>Paula Moshier & Joyce J. Fitzpatrick<br>(2024)	USA	Descriptive cross-sectional study	To develop safety-related knowledge for incorporation into evidence-based nursing practice by measuring nurse leaders' perceptions of just culture in the hospital setting.	Hospital setting	✓	✓	✓	✓
Motivators and inhibitors of nurses' speaking up behaviours: A descriptive qualitative study<br><br>Lee, S. E.; Dahinten, V. S.; Ji, H.; Kim, E. & Lee, H.<br>(2022)	South Korea	Descriptive qualitative study	The aim of this study was to identify factors that motivate or inhibit nurses' speaking up for patient safety.	"15 nurses from four Korean hospitals" / "direct care nurses who worked in medical-surgical units or the emergency departments at four tertiary hospitals in metropolitan areas of South Korea."	✓	✓	✓	✓
A study of error reporting by nurses: the significant impact	USA	Cross-sectional descriptive study	The aim of this study was to understand how the nursing team dynamics of leader inclusiveness,	"The setting for the study was a large academic medical centre in the southeastern United States." / "A convenience	✓	✓	✓	✓

Table S2

Summary of included studies according to study characteristics and extracted thematic information

Title<br>Author(s)<br>(Year)	Country of origin	Type of study	Goal(s)	Context	Nurse managers and psychological safety	Nurse managers and patient safety culture	Relationship between psychological safety and patient safety culture	Facilitators and barriers
of nursing team dynamics<br><br>Munn, L. T.; Lynn, M. R.; Knafl, G. J.; Willis, T. S.; Jones, C. B. (2023)			safety climate and psychological safety affected the willingness of hospital nurses to report errors.	sample of nurses and nurse managers employed by the hospital was recruited from 53 patient care units within the hospital."				
Organizational Silence as a Mediator Between Ethical Nursing Competence and Perceptions of Open Disclosure in Patient Safety Incidents (PSIs)<br><br>Yuna Kim, Youn-Jung Son, Sun Joo Jang, Haeyoung Lee (2026)	South Korea	Cross-sectional, theory-driven correlational survey	To test organizational silence's mediating role between nurses' ethical competence and perceptions of open disclosure in PSIs.	General and tertiary hospitals	✓	✓	✓	✓
Psychological Safety as a Mediator of the Relationship Between Inclusive Leadership and Nurse Voice Behaviors and Error Reporting	South Korea	Correlational cross-sectional web-based survey	To examine psychological safety as a mediator of the relationship between inclusive leadership and nurses' voice behaviors and error reporting.	Medical/surgical units in three tertiary general hospitals	✓	✓	✓	✓

Table S2

Summary of included studies according to study characteristics and extracted thematic information

Title<br>Author(s)<br>(Year)	Country of origin	Type of study	Goal(s)	Context	Nurse managers and psychological safety	Nurse managers and patient safety culture	Relationship between psychological safety and patient safety culture	Facilitators and barriers
Seung Eun Lee, V. Susan Dahinten (2021)								
Patient safety ratings of hospitals by registered nurses, managers, and executives: A retrospective analysis of patient safety culture data<br><br>DeAnna Jan Emory, Thomas A. Kippenbrock (2025)	USA	Retrospective analysis / Exploratory quantitative study	Understand differences in perceptions of patient safety culture among registered nurses, managers, and executives, to identify gaps that impact safety initiatives.	Hospital	✓	✓	✓	✓
The ideal-reality gap: a qualitative study of nurse middle managers' perspectives on speaking up for patient safety<br><br>Jiahui Dong, Li Du, Jiangtao Xu, Jing Li, Jingze Tao, Fang Wang (2026)	China	Descriptive qualitative study using semi-structured interviews	To explore nurse middle managers' perceptions and recommendations regarding nurses' Speaking Up for Patient Safety (SUPS)	Tertiary Grade A hospital	✓	✓	✓	✓

Table S2

Summary of included studies according to study characteristics and extracted thematic information

Title<br>Author(s)<br>(Year)	Country of origin	Type of study	Goal(s)	Context	Nurse managers and psychological safety	Nurse managers and patient safety culture	Relationship between psychological safety and patient safety culture	Facilitators and barriers
The dark side of nurse-manager leadership: toxic leadership behaviours, adverse-event reporting, and care quality—an interpretive descriptive study<br><br>Mohammed Alshmemri (2026)	Saudi Arabia	Qualitative study; interpretive description.	Primary Aim:<br><br>To generate theory-informed and practice-relevant explanations of how toxic nurse manager leadership behaviours influence adverse-event reporting and care quality in hospital settings.	Hospital setting<br><br>staff nurses (n = 26), nurse managers (n = 7), and quality officers (n = 2) (total N = 35),	✓	✓	✓	✓
Association between abusive supervision and nurses' withholding voice about patient safety: the roles of impression management motivation and speak up-related climate<br><br>Zhi-Ying Li, et al. (2024)	China	Quantitative cross-sectional survey study	To explore the relationship between abusive supervision by nurse managers, impression management motivation, speak up-related climate, and nurses' withholding voice about patient safety; to examine how impression management motivation mediates and speak up-related climate moderates this relationship	Taizhou Hospital, Zhejiang Province, China; 419 clinical nurses recruited via convenience sampling between November 2022 and January 2023; single tertiary public hospital with 1,413 clinical nurses	✓	✓	✓	✓
Leader Behavioral Integrity for Safety, Priority of Safety, Psychological	Belgium	Quantitative observational study; multilevel survey study using	To examine how head nurse behavioral integrity for safety influences team priority of safety, psychological safety, and patient safety outcomes	Four Belgian hospitals; 54 nursing departments/teams.	✓	✓	✓	✓

Table S2

Summary of included studies according to study characteristics and extracted thematic information

**Title Author(s) (Year)**	**Country of origin**	**Type of study**	**Goal(s)**	**Context**	**Nurse managers and psychological safety**	**Nurse managers and patient safety culture**	**Relationship between psychological safety and patient safety culture**	**Facilitators and barriers**
Safety, and Patient Safety Leroy et al. (2012)		structural equation modeling	(reported treatment errors).					
Global Perspectives on Patient Safety: The Central Role of Nursing Management Anders (2025)	USA	Narrative review	This narrative review synthesizes global evidence on how nursing management practices, particularly leadership, staffing, and safety culture, influence patient safety outcomes across diverse health systems.	The context included all healthcare settings (acute care, long-term care, community health) across all geographic regions and healthcare system types.	✓	✓	✓	✓

Note. ✓ indicates that information related to this item was identified in the article.
